# Traditional Chinese medicine improves performance and intestinal health in laying hens under acute and chronic heat stress by modulating ileal metabolic functions

**DOI:** 10.1016/j.psj.2026.107056

**Published:** 2026-05-01

**Authors:** Zi Mei, Haobo Zhou, Kunyuan Liu, Chaoyang Gao, Hao Du, Zheya Sheng, Yanzhang Gong

**Affiliations:** Key Laboratory of Agricultural Animal Genetics, Breeding and Reproduction, Ministry of Education; College of Animal Science and Technology and College of Veterinary Medicine, Huazhong Agricultural University, Wuhan, Hubei Province, 430070, PR China

**Keywords:** Heat stress, Laying hen, Traditional Chinese medicine, Gut microbiome, Metabolomics

## Abstract

Heat stress (HS) represents a significant challenge in poultry production, impairing thermoregulation, intestinal function, and productive performance. This study utilized acute (6 h) and chronic (14 d) HS models at 36°C in laying hens to characterize stage-dependent responses and evaluate the protective effects of a ten-ingredient traditional Chinese medicine (TCM) formulation. Both acute and chronic HS significantly increased rectal temperature and respiratory rate. Egg production declined by approximately 18% following acute HS and was further compromised under chronic exposure, along with reduced eggshell strength and weight. Dietary TCM supplementation (0.5%) alleviated physiological stress and partially restored laying performance, with more pronounced recovery observed under chronic HS. Serum analysis and histopathology indicated that TCM attenuated HS-induced impairment of ileal barrier function. Metabolomic profiling revealed stage-dependent responses: acute HS primarily disturbed redox balance, whereas chronic HS induced broader remodeling related to energy and nutrient utilization. TCM supplementation modulated metabolic functions to support immediate stress buffering under acute HS while stabilizing long-term energy support and intestinal capacity under chronic HS. Metagenomic analysis indicated that TCM selectively promoted microbial groups related to intestinal metabolism and nutrient utilization, aligning with metabolomic findings. Correlation analyses linked these TCM-associated microbial and metabolic signatures with improved thermoregulatory responses, oxidative status, and intestinal barrier indicators. Collectively, these results demonstrate that TCM supplementation enhances heat resilience in laying hens through stage-dependent modulation of the gut microbiota-metabolome axis, supporting its application as a nutritional strategy to maintain productivity under thermal challenge.

## Introduction

Heat stress (HS) is one of the most critical environmental challenges affecting poultry production, particularly in regions with high ambient temperatures and increasing climate variability (Kim et al., 2024; Ncho, 2025; Oluwagbenga and Fraley, 2023). When environmental temperatures exceed the thermoneutral zone, laying hens experience impaired thermoregulation, resulting in elevated rectal temperature, increased respiratory rate, and pronounced endocrine disturbances (Kim, et al., 2021, 2024, 2025). These physiological responses are frequently accompanied by metabolic dysfunction, including altered glucose and cholesterol homeostasis, leading to reduced egg production, compromised eggshell quality, and substantial economic losses in commercial operations worldwide (Mei, et al., 2025; Nanto-Hara, et al., 2023). Consequently, heat stress not only threatens animal welfare but also leads to substantial economic losses in commercial laying hen operations worldwide.

In recent years, the intestine has been recognized as a primary target of HS-induced injury in poultry (Huang, et al., 2024; Ncho, 2025; Wickramasuriya, et al., 2022). Elevated temperatures disrupt intestinal morphology and barrier function, as evidenced by villus shortening and the downregulation of tight junction proteins, which increases epithelial permeability and systemic inflammation (Kikusato and Toyomizu, 2023; Ncho, 2025). These alterations are closely associated with oxidative stress and profound shifts in gut microbiota composition, which can compromise energy metabolism and physiological homeostasis (Ahmad, et al., 2022; Olayiwola and Adedokun, 2025; Zhu, et al., 2019). Thus, the intestine represents a critical interface linking environmental thermal stress to systemic redox balance and productive performance in laying hens.

Nutritional interventions have been widely explored to mitigate HS, among which traditional Chinese medicine (TCM) has received increasing attention due to its multi-target synergistic effects and minimal risk of residues (Fayed, et al., 2024; Mahasneh, et al., 2024; Ye, et al., 2022). Compared with synthetic additives, TCM is generally characterized by wide availability, low toxicity, and minimal risk of residues or antimicrobial resistance. Previous studies have shown that individual herbal components, such as baicalin, puerarin, and astragalus polysaccharides, can alleviate oxidative stress, modulate inflammatory responses, and enhance immune function in heat-stressed poultry (Cong, et al., 2017; Liu, et al., 2025; Sampath, et al., 2025). Moreover, compound TCM formulations, developed according to traditional therapeutic principles, may exert synergistic effects by simultaneously supporting antioxidant capacity, immune regulation, and intestinal function, while also modulating gut microbial communities (Wang, et al., 2025; Zhang, et al., 2025).

Despite these advances, the extent to which TCM modulates host responses to acute versus chronic heat stress, particularly with respect to gut microbiota-associated metabolic alterations, remains incompletely understood. In the present study, a TCM formulation was evaluated in laying hens subjected to acute (6 h) and chronic (14 d) heat stress. We assessed its effects on physiological stress responses, laying performance, and intestinal barrier function. In addition, metagenomic and metabolomic analyses were conducted to explore TCM-associated changes in gut microbial features and metabolic profiles under different heat stress conditions. Together, this study provides experimental evidence supporting the potential application of TCM as a nutritional strategy to improve heat stress resilience and maintain productive performance in laying hens.

## Materials and methods

### Statement of institutional animal care and use committee

The experimental protocol and all animal handling procedures were strictly reviewed and approved by the Experimental Animal Management and Ethics Committee of Huazhong Agricultural University (Approval No. HZAUCH-2025-0036). The study was conducted in accordance with the principles of the International Guide for the Care and Use of Laboratory Animals to ensure animal welfare and minimize distress.

### TCM preparation and diet

The traditional Chinese medicine (TCM) formulation used in this study was a compound mixture consisting of ten ingredients: *Gypsum Fibrosum* (gypsum), *Coptidis Rhizoma* (Coptis rhizome), *Gardeniae Fructus* (gardenia fruit), A*tractylodis Macrocephalae Rhizoma* (largehead atractylodes rhizome), *Paeoniae Radix Alba* (white peony root), *Rehmanniae Radix* (rehmannia root), *Moutan Cortex* (tree peony bark), *Anemarrhenae Rhizoma* (anemarrhena rhizome), *Glycyrrhizae Radix et Rhizoma* (licorice root), and sodium bicarbonate. All components were obtained from a standardized supplier, mixed in defined proportions, ground into a fine powder capable of passing a 200-mesh sieve, and incorporated into the basal diet at 0.5% of total feed weight (w/w).

### Experimental design and heat stress model

Eighty 29-week-old Xinhua No. 2 laying hens were used in this study. Prior to the formal experiment, birds were acclimated for 14 days in a controlled environmental facility at a thermoneutral temperature (23 ± 1°C) and 60-70% relative humidity, with a photoperiod of 15 h light and 9 h dark.

**Fig. 1:**
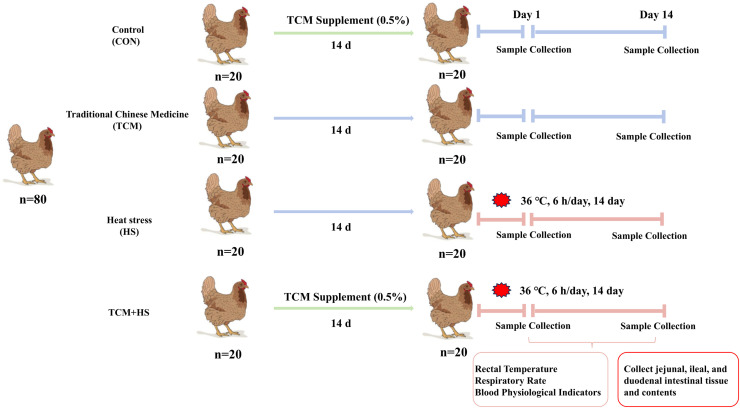
Schematic of experimental design showing four treatment groups (CON, TCM, HS, TCM.HS).

Hens were randomly assigned to one of four treatment groups (n = 20 per group): control (CON), TCM-supplemented (TCM), heat stress (HS), and TCM supplementation plus heat stress (TCM.HS). The CON group was maintained under thermoneutral conditions (23 ± 1°C) throughout the experiment without TCM supplementation. Hens in the TCM and TCM.HS groups received dietary TCM supplementation for 14 days prior to heat stress exposure. During this pre-treatment period, all hens were housed under thermoneutral conditions. Following the pre-treatment period, hens in the HS and TCM.HS groups were transferred to a heat stress chamber and exposed daily to 36 ± 1°C for 6 h for 14 consecutive days, while the CON and TCM groups remained under thermoneutral conditions (Fig. 1).

### Sample collection

Rectal temperature (RT) and respiratory rate (RR) were measured in hens from the CON, TCM, HS, and TCM.HS groups at two corresponding experimental time points: Day 1 (defined as acute heat stress, AHS) and Day 14 (defined as chronic heat stress, CHS). For the HS and TCM.HS groups, measurements were performed immediately after the daily 6-h heat exposure, whereas for the CON and TCM groups, measurements were conducted at the same time of day under thermoneutral conditions. Rectal temperature was measured using a digital thermometer inserted approximately 3 cm into the cloaca, and respiratory rate was determined by counting thoracic movements over a 60-s period. At the same time points, blood samples were collected from the brachial vein, centrifuged at 3,000 × g for 15 min at 4°C to obtain serum, and stored at −80°C until analysis.

For egg production performance assessment, eggs from all remaining hens in the CON, TCM, HS, and TCM.HS groups were collected daily throughout the 14-day heat stress period. Egg weight was determined using an electronic balance, shell strength was measured using a shell strength tester, and Haugh units were assessed using an egg quality analyzer according to the manufacturer’s instructions.

On Days 1 and 14, immediately following the 6-h heat stress exposure, five hens were randomly selected from each group and humanely euthanized. Segments of the duodenum, jejunum, and ileum were collected. Intestinal tissues for histological analysis were fixed in 4% paraformaldehyde, whereas tissues for molecular analyses were snap-frozen in liquid nitrogen and stored at −80°C. Intestinal contents were also rapidly frozen in liquid nitrogen and stored at −80°C for subsequent molecular and omics analyses.

### Biochemical and oxidative stress assays

On heat stress Days 1 and 14, immediately following the daily 6-h heat stress exposure, blood samples were collected from hens in the CON, TCM, HS, and TCM.HS groups, and serum was separated for physiological parameter analysis. Serum glucose (GLU) and total cholesterol (TCHO) levels were measured using a fully automated biochemical analyzer (BX-4000, Sysmex, Japan). Serum corticosterone (CORT), heat shock protein 70 (HSP70), lactate dehydrogenase (LDH), diamine oxidase (DAO), and d-lactic acid (DLA) concentrations were determined using commercial ELISA kits (Nanjing Jiancheng Biological Engineering Institute, China) according to the manufacturers’ instructions.

### Histological analysis

Intestinal tissues collected from hens in the CON, TCM, HS, and TCM.HS groups on Days 1 and 14 were fixed in 4% paraformaldehyde for 24 h, dehydrated through a graded ethanol series, cleared in xylene, and embedded in paraffin. Sections (6 μm) were prepared using a rotary microtome (Leica RM2235, Germany) and stained with hematoxylin and eosin (H&E). The duodenum, jejunum, and ileum from the CON, TCM, HS, and TCM.HS groups were examined and photographed using an optical microscope (Olympus BX53, Japan). Quantitative analysis of villus height and crypt depth was performed using ImageJ software (NIH, USA).

### Gene expression analysis

For gene expression, total RNA was extracted from ileal tissues collected from hens in the CON, TCM, HS, and TCM.HS groups using TRIzol reagent (TIANGEN, China). After verifying RNA quality and concentration, cDNA was synthesized using a reverse transcription kit (TaKaRa, Japan). Quantitative real-time PCR (qPCR) was performed using SYBR Green Master Mix (Vazyme, China) to quantify the mRNA expression of tight junction proteins (*ZO-1, CLDN1, OCLN*) and *HSP70*, with *β-actin* as the internal reference. The primer sequences used for quantitative real-time PCR (qPCR) are listed in Table 1.

**Table 1:** Primer sequences used for quantitative real-time PCR.

*Gene*	Primer sequences (5′ to 3′)	Accession No.
*β‐actin*	f-TTGTTGACAATGGCTCCGGT	NM_205518.1
	r-TCTGGGCTTCATCACCAACG	
*Occludin*	f-CGCAGATGTCCAGCGGTTACT	NM_205128.1
	r-CAGAGCAGGATGACGATGAGGAA	
*ZO‐1*	f-CCACTGCCTACACCACCATCTC	XM_015278975.4
	r-CGTGTCACTGGGGTCCTTCAT	
*Claudin‐1*	f-GCATGGAGGATGACCAGGTGA	NM_001013611.2
	r-GAGCCACTCTGTTGCCATACCAT	
*HSP70*	f-CGTCAGTGCTGTGGACAAGAGTA	NM_001006685.2
	r-CCTATCTCTGTTGGCTTCATCCT	

### Antioxidant index measurement in the ileum

Ileal tissue samples were accurately weighed and homogenized in ice-cold phosphate-buffered saline (PBS) at a ratio of 1:9 (w/v) using a tissue homogenizer. The homogenates were centrifuged at 12,000 × g for 10 min at 4°C, and the supernatants were collected for antioxidant analysis. The levels of malondialdehyde (MDA), total antioxidant capacity (T-AOC), and the activity of superoxide dismutase (SOD) in ileal homogenates were determined using commercial assay kits (Nanjing Jiancheng Bioengineering Institute, China) according to the manufacturer’s instructions. Protein concentrations were measured using a bicinchoninic acid (BCA) protein assay kit, and antioxidant indices were normalized to total protein content.

### Metagenomic sequencing and bioinformatics

For metagenomic analysis, ileal contents were collected from three hens per group in the CON, HS, and TCM.HS groups at two corresponding experimental time points: Day 1 (defined as acute heat stress, AHS) and Day 14 (defined as chronic heat stress, CHS). Microbial genomic DNA was extracted from ileal contents using a commercial DNA extraction kit following the manufacturer’s instructions. DNA concentration and purity were assessed using a Qubit 4 fluorometer and agarose gel electrophoresis, respectively. Sequencing libraries with an average insert size of approximately 350 bp were constructed and sequenced on the Illumina NovaSeq platform (paired-end 150 bp). Raw reads were quality-filtered using fastp, and host-derived sequences were removed by aligning reads to the chicken reference genome using Bowtie2. All metagenomic libraries were prepared and sequenced in a single batch to minimize potential batch effects. Normalized read counts were used for downstream analyses without additional batch correction. High-quality reads were assembled de novo using MEGAHIT, and open reading frames (ORFs) were predicted using MetaGeneMark. Redundant sequences were removed using CD-HIT. Taxonomic annotation was performed by aligning predicted protein sequences against the NCBI non-redundant (NR) database using DIAMOND, and taxonomic assignments were determined using the lowest common ancestor (LCA) algorithm implemented in MEGAN.

Principal component analysis (PCA) was conducted as an unsupervised multivariate approach based on normalized abundance profiles to visualize overall microbial community structure and evaluate differences among experimental groups. Microbial diversity analyses and identification of differential taxa and functional profiles were performed using MetaGenomeSeq and LEfSe. Functional annotation was conducted using the KEGG, eggNOG, CAZy, VFDB, and PHI databases, and antibiotic resistance genes were identified using the CARD database via the RGI pipeline.

### Untargeted metabolomics of ileal contents

For untargeted metabolomics analysis, ileal contents were collected from three hens per group at two corresponding experimental time points, Day 1 (defined as acute heat stress, AHS) and Day 14 (defined as chronic heat stress, CHS), from the CON, HS, and TCM.HS groups. Approximately 20 mg of ileal content was extracted with 400 μL methanol-water solution (7:3, v/v) containing an internal standard. Samples were vortexed, sonicated, and centrifuged, and the supernatants were subjected to LC-MS analysis in information-dependent acquisition (IDA) mode using Analyst TF 1.7.1 software (Sciex, Canada). This approach allowed for the detection and quantification of a broad range of small-molecule metabolites, including amino acids, lipids, sugars, and other compounds, to comprehensively characterize metabolic profiles among groups.

Metabolite abundances were normalized to internal standards, log₂-transformed, and mean-centered prior to multivariate analysis. Principal component analysis (PCA) was performed as an unsupervised method to visualize global metabolic patterns and assess group separation. Orthogonal partial least squares–discriminant analysis (OPLS-DA) models were constructed using MetaboAnalystR and validated by 200 permutation tests. Differential metabolites were identified based on variable importance in projection (VIP > 1), |fold change| > 2, and *P* < 0.05 (Student’s t-test). Identified differential metabolites were further imported into MetaboAnalyst 6.0 for pathway enrichment analysis. Spearman's correlation analysis was employed to evaluate the association between differential metabolites and physiological indicators.

### Statistical analysis

Statistical analyses were performed using SPSS 26.0 (IBM, USA) and GraphPad Prism 8.0.2. Data are presented as mean ± SEM, with each individual hen considered as the experimental unit. Data were tested for normality and homogeneity of variance prior to statistical analysis. Student’s t-tests were used for direct two-group comparisons where appropriate, such as comparisons between the CON and HS groups, or between the HS and TCM.HS groups in gut microbiota and metabolomics analyses. For outcomes involving all four experimental groups (CON, TCM, HS, and TCM.HS), including egg quality, physiological parameters, and intestinal measurements, one-way analysis of variance (ANOVA) was performed, followed by Tukey’s post hoc test to identify significant differences among groups. A P value < 0.05 was considered statistically significant.

## Results

### Traditional Chinese medicine (TCM) supplementation exerts systemic protective effects against acute and chronic heat stress

**Fig. 2:**
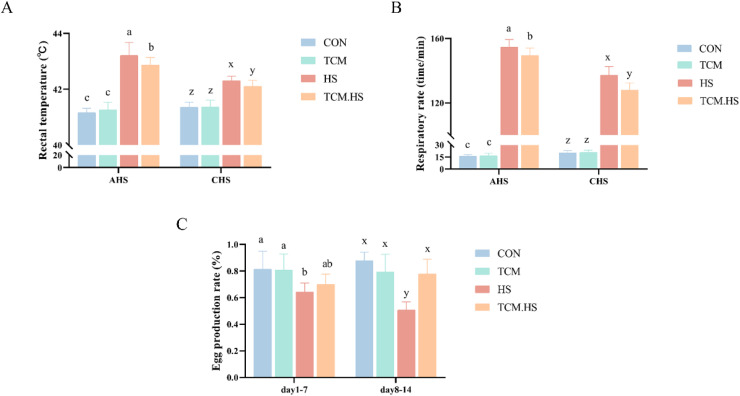
Effects of traditional Chinese medicine supplementation on thermoregulation and laying performance of laying hens under heat stress. (A) Rectal temperature. (B) Respiratory rate. (C) Laying rate. In the same rank, a, b, c values with different small letter superscripts mean significant difference (*P* < 0.05).

Traditional Chinese medicine (TCM) supplementation alleviates physiological and productive impairments induced by acute and chronic heat stress. To characterize heat stress-induced physiological disturbances, a chicken heat-stress model was established at 36°C in a controlled climate chamber. Both acute (6 h, AHS) and chronic (6 h/day for 14 days, CHS) heat stress models were evaluated. Compared with non-heat-stressed controls, rectal temperature (RT) and respiratory rate (RR) were significantly elevated in both AHS and CHS groups (Fig. 2A and B).

Laying performance was concurrently impaired. In the heat-stressed group, egg production decreased from 81.4% to 64.3% during days 1-7 of heat stress and further declined to 50.7% during days 8-14. Significant reductions in laying rate, egg weight, and eggshell strength were also observed (Fig. 2C; Table 2). Dietary supplementation with TCM significantly mitigated these effects, with RT and RR reduced and egg production recovering to 77.8% in the TCM.HS group (Fig. 2A-B). Furthermore, TCM supplementation partially improved productive performance, restoring laying rate during days 8-14 of CHS and attenuating CHS-induced reductions in egg weight and eggshell strength (Fig. 2C; Table 2).

**Table 2:** Effects of traditional Chinese medicine (TCM) supplementation on egg quality traits in laying hens under heat stress.

Item	Treatment			
	CON	TCM	HS	TCM.HS
Egg weight (g)				
day1	58.10 ± 4.23	60.68 ± 3.43	57.28 ± 3.89	57.77 ± 4.21
day7	57.333 ± 3.01^a^	58.02 ± 4.25^a^	52.86 ± 1.89^b^	57.41 ± 4.10^a^
day14	58.63 ± 4.68^a^	58.89 ± 3.95^a^	53.40 ± 2.41^b^	57.86 ± 3.07^b^
Eggshell strength (kg/cm^2^)				
day1	4.27 ± 00.98	4.23 ± 0.96	3.94 ± 1.09	4.23 ± 0.83
day7	4.46 ± 0.87^a^	4.39 ± 1.00^a^	3.20 ± 0.80^b^	4.26 ± 0.83^a^
day14	4.44 ± 0.87^a^	4.49 ± 1.11^a^	3.39 ± 0.61^b^	4.40 ± 0.74^b^
Egg yolk color				
day1	5.96 ± 1.30	5.88 ± 0.90	5.51 ± 1.56	5.30 ± 1.10
day7	6.18 ± 1.19	5.94 ± 1.01	5.32 ± 0.93	5.32 ± 1.29
day14	4.93 ± 1.00	5.41 ± 0.93	5.03 ± 1.27	5.29 ± 0.87
Haugh unit				
day1	93.22 ± 4.72	90.93 ± 5.80	92.28 ± 6.04	90.58 ± 4.13
day7	90.64 ± 5.80	89.75 ± 4.51	85.93 ± 10.18	92.15 ± 5.57
day14	89.77 ± 4.40	88.82 ± 5.66	85.34 ± 6.50	87.99 ± 6.32

Notes: Values are presented as mean ± SEM. Different lowercase letters (a-c) within the same row indicate significant differences among treatment groups at the same time point (*P* < 0.05).

### Dietary TCM alleviates heat stress-induced alterations in serum physiological indicators

**Fig. 3:**
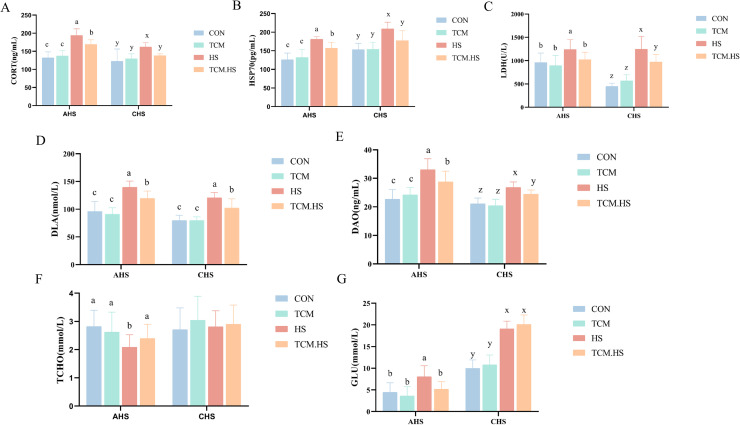
Effects of traditional Chinese medicine supplementation on serum stress indicators, intestinal permeability, and metabolic parameters of laying hens under heat stress. (A) CORT. (B) HSP70. (C) LDH. (D) DLA. (E) DAO. (F) TCHO. (G) GLU. In the same rank, a, b, c values with different small letter superscripts mean significant difference (*P* < 0.05). HSP70: heat shock protein 70, CORT: corticosterone, LDH: lactate dehydrogenase, DLA: d-lactic acid, DAO: diamine oxidase, GLU: glucose, TCHO: total cholesterol.

Serum stress-related indicators, including heat shock protein 70 (HSP70), corticosterone (CORT), and lactate dehydrogenase (LDH), were significantly elevated in both AHS and CHS groups compared with controls (Fig. 3A-C). Serum d-lactic acid (DLA) and diamine oxidase (DAO) concentrations were also increased under both heat stress conditions (Fig. 3D-E). Regarding metabolic parameters, serum glucose (GLU) and total cholesterol (TCHO) showed differential responses between AHS and CHS. Following AHS, GLU levels were increased while TCHO levels were reduced, whereas under CHS, GLU remained elevated and TCHO levels showed no significant difference from controls (Fig. 3F-G). Dietary TCM supplementation significantly mitigated these effects, reducing serum concentrations of HSP70, CORT, LDH, DLA, and DAO (Fig. 3A-E). Under CHS, HSP70 and CORT levels were restored to pre-stress values in the TCM-supplemented group. Consistent with the differential metabolic responses to AHS and CHS, GLU and TCHO levels were restored under TCM.AHS, whereas CHS and TCM-CHS groups showed no significant differences in TCHO and GLU (Fig. 3F-G).

### Histological and transcriptional evidence of heat stress-induced ileal barrier disruption and its attenuation by TCM supplementation

**Fig. 4:**
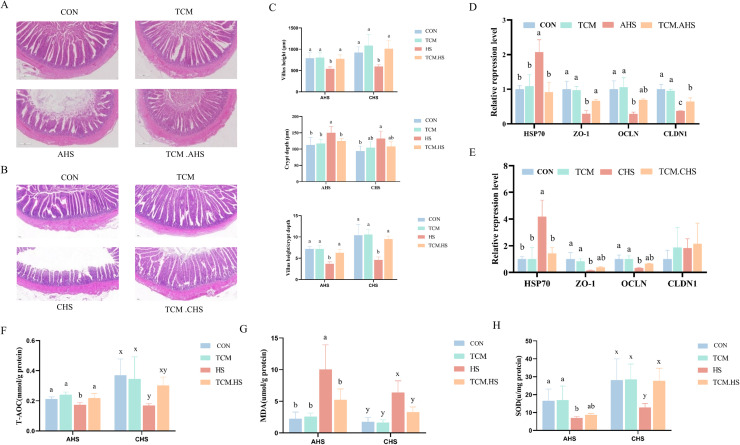
Effects of traditional Chinese medicine supplementation on ileal morphology, gene expression, and oxidative status of laying hens under heat stress. (A-C) Ileal morphology, including representative H&E staining sections (A, B) and morphometric parameters (C). (D-E) Relative mRNA expression of *ZO-1, OCLN, CLDN1*, and *HSP70* under AHS (D) and CHS (E). (F) T-AOC. (G) MDA. (H) SOD. In the same rank, a, b, c values with different small letter superscripts mean significant difference (*P* < 0.05). AHS: acute heat stress, CHS: chronic heat stress, VH: villus height, CD: crypt depth, VH/CD: villus height-to-crypt depth ratio, ZO-1: zonula occludens-1, OCLN: occludin, CLDN1: claudin-1, HSP70: heat shock protein 70, T-AOC: total antioxidant capacity, MDA: malondialdehyde, SOD: superoxide dismutase.

Heat stress significantly elevated d-lactic acid (DLA) and diamine oxidase (DAO) levels, indicating ileal barrier dysfunction. Histopathological analysis revealed that both acute (AHS) and chronic (CHS) heat stress impaired ileal morphology, as shown by villus shortening, increased crypt depth, and a reduced villus height-to-crypt depth ratio, whereas duodenum and jejunum morphology remained largely unchanged (Fig. 4A-C; Supplementary Table 1). Consistent with these morphological changes, ileal transcriptional levels of the tight junction protein *ZO-1* were significantly decreased after both AHS and CHS, while *CLDN1* expression was reduced only after AHS. Additionally, *HSP70* was markedly upregulated under both heat stress conditions (*P* < 0.05; Fig. 4D-E). Heat stress also disrupted ileal oxidative status, as evidenced by decreased superoxide dismutase (SOD) activity and total antioxidant capacity (T-AOC), alongside increased malondialdehyde (MDA) levels (*P* < 0.05; Fig. 4F-H). Dietary supplementation with TCM improved ileal histomorphology and antioxidant capacity. Villus height and the villus height/crypt depth ratio were significantly increased, while *HSP70* and MDA levels were reduced in both TCM-AHS and TCM-CHS groups (Fig. 4A-H). Interestingly, TCM effects differed between AHS and CHS conditions: crypt depth, *ZO-1, CLDN1*, and T-AOC were significantly improved under AHS, whereas SOD activity was significantly enhanced only in TCM-CHS compared with CHS (Fig. 4C-G).

### Gut microbiome alterations under acute and chronic heat stress

**Fig. 5:**
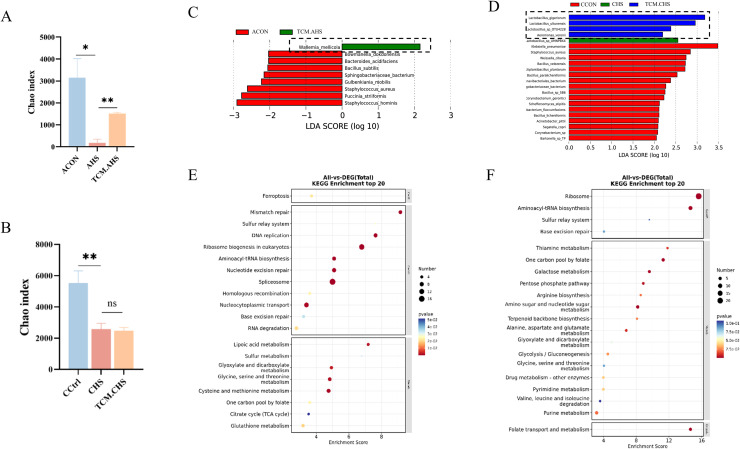
Effects of traditional Chinese medicine supplementation on ileal microbial diversity and functional potential of laying hens under heat stress. (A-B) Microbial richness (Chao1 index) under AHS (A) and CHS (B). (C-D) LEfSe analysis identifying characteristic microbial taxa in TCM.AHS (C) and TCM.CHS (D). (E-F) Functional enrichment analysis of microbial biomarkers in TCM.AHS (E) and TCM.CHS (F). In the same rank, a, b, c values with different small letter superscripts mean significant difference (*P* < 0.05). LEfSe: linear discriminant analysis effect size.

Metagenomic sequencing was performed to characterize ileal microbial communities across experimental groups. Principal component analysis (PCA) based on species abundance showed clear separation between control and heat-stressed (HS) groups, with TCM-treated groups positioned intermediately, indicating partial restoration of heat stress-induced microbial dysbiosis (Fig. S1). Both acute and chronic heat stress significantly reduced microbial richness, as reflected by decreased Chao1 indices compared with controls (*P* < 0.05) (Fig. 5A-B). TCM supplementation significantly increased microbial richness under acute heat stress, whereas no significant effect was observed under chronic heat stress (Fig. 5A-B).

LEfSe analysis identified characteristic taxa associated with TCM supplementation under different heat stress conditions. Under acute heat stress, Wallemia mellicola was enriched in the TCM.AHS group, while multiple Lactobacillus species were characteristic of the TCM.CHS group (Fig. 5C-D). Functional enrichment analysis revealed that W. mellicola in the TCM.AHS group was mainly associated with genetic information processing and metabolic pathways, including mismatch repair, lipoic acid metabolism, glycine, serine and threonine metabolism, and one-carbon pool by folate (Fig. 5E). In the TCM.CHS group, characteristic taxa were predominantly enriched in metabolic pathways, with some overlap such as glycine, serine and threonine metabolism and one-carbon pool by folate, whereas thiamine metabolism was uniquely enriched under chronic heat stress (Fig. 5F).

**Fig. 6:**
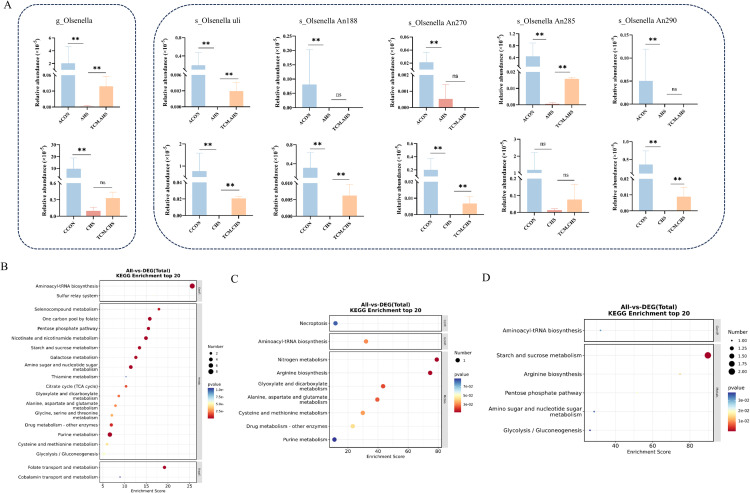
Effects of traditional Chinese medicine supplementation on the abundance and functional potential of Olsenella species in the ileum of heat-stressed laying hens. (A) Relative abundance of Olsenella species. (B-C) Functional enrichment analysis of Olsenella strains enriched in TCM.AHS (B) and TCM.CHS (C). (D) Functional enrichment analysis of Olsenella uli. * indicates *P* < 0.05, ** indicates *P < 0.01*.

Several *Olsenella* species, markedly reduced under both acute and chronic heat stress, exhibited partial recovery following TCM supplementation (Fig. 6A). Olsenella uli was consistently restored under both conditions (Fig. 6A). Functional enrichment analysis revealed distinct metabolic profiles: strains enriched under TCM.AHS were associated with Selen compound metabolism, one-carbon pool by folate, and glycine, serine and threonine metabolism, whereas strains enriched under TCM.CHS were mainly involved in nitrogen metabolism, arginine biosynthesis, and glyoxylate and dicarboxylate metabolism (Fig. 6B-C). Notably, *Olsenella uli*, restored under both stress conditions, was enriched in starch and sucrose metabolism, arginine biosynthesis, and glycolysis/gluconeogenesis, suggesting a conserved metabolic role associated with TCM-mediated microbial recovery (Fig. 6D).

### Metabolomic reprogramming under acute and chronic heat stress

**Fig. 7:**
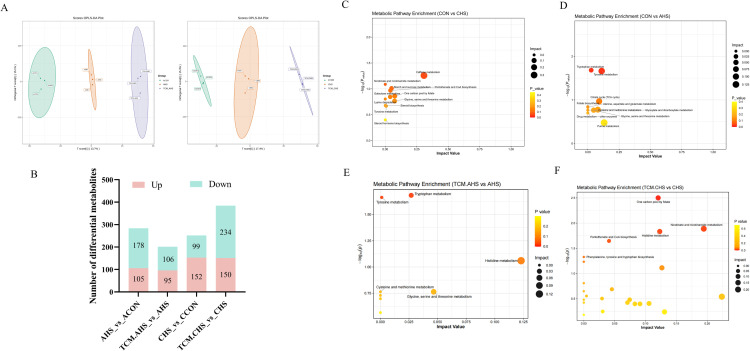
Effects of traditional Chinese medicine supplementation on the ileal metabolome of laying hens under heat stress. (A) OPLS-DA score plot of ileal metabolites. (B) Number of differential metabolites. (C-D) Pathway enrichment analysis of differential metabolites under AHS (C) and CHS (D) compared with controls. (E-F) Pathway enrichment analysis of differential metabolites in TCM.AHS (E) and TCM.CHS (F) compared with respective heat-stressed groups.

Metabolomic profiling of ileal contents revealed pronounced remodeling of the ileal metabolome under both acute (AHS) and chronic heat stress (CHS) conditions. Consistently, principal component analysis (PCA) showed an overall separation of metabolic profiles among treatment groups under both AHS and CHS conditions (Fig. S2). Partial least squares-discriminant analysis (OPLS-DA) demonstrated clear separation among experimental groups, indicating substantial metabolic perturbations caused by heat stress (Fig. 7A). Differential metabolites were identified based on variable importance in projection (VIP > 1), |fold change| > 2, and *P* < 0.05 (Fig. 7B). Untargeted metabolomic analysis showed distinct pathway enrichment patterns in response to acute and chronic heat exposure. In the AHS group, differential metabolites relative to controls were primarily enriched in amino acid-related pathways, including tryptophan metabolism, tyrosine metabolism, and glycine, serine and threonine metabolism (Fig. 7C). In contrast, CHS induced broader and more sustained alterations, affecting one-carbon pool by folate, glycine, serine and threonine metabolism, histidine metabolism, and nicotinate and nicotinamide metabolism (Fig. 7D).

**Fig. 8:**
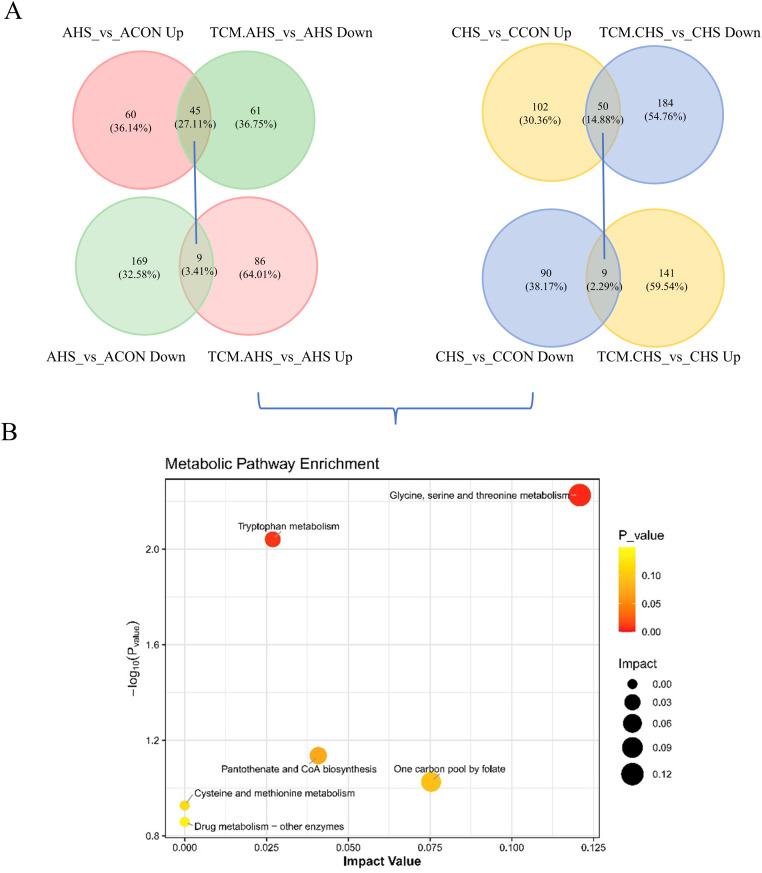
Intersection analysis of differential metabolites and core metabolic pathways modulated by traditional Chinese medicine under heat stress. (A) Identification of intersected metabolites consistently altered by heat stress and reversed following TCM supplementation. (B) Functional enrichment analysis of the core metabolic pathways mitigated by TCM under both acute and chronic heat stress.

To evaluate the regulatory effects of TCM, pathway enrichment analyses were further performed by comparing TCM-treated groups with their respective heat-stressed counterparts. Under acute heat stress, TCM supplementation primarily modulated amino acid metabolic pathways, including tryptophan metabolism and glycine, serine and threonine metabolism, partially attenuating AHS-induced perturbations in these pathways (Fig. 7E). Under chronic heat stress, TCM exerted more pronounced effects on pathways related to metabolic homeostasis, with significant enrichment observed in one-carbon pool by folate, pantothenate and CoA biosynthesis, cysteine and methionine metabolism, and glycine, serine and threonine metabolism, suggesting an association with improved metabolic stability and energy-related processes (Fig. 7F). Importantly, intersection analysis of differential metabolites between control versus heat stress and TCM versus heat stress comparisons identified a subset of metabolites consistently altered by heat stress and reversed following TCM supplementation (Fig. 8A). Functional enrichment of these intersected metabolites revealed that glycine, serine and threonine metabolism, tryptophan metabolism, one-carbon pool by folate, and cysteine and methionine metabolism represented the core metabolic pathways mitigated by TCM under both acute and chronic heat stress conditions (Fig. 8B). These findings indicate that TCM does not globally reshape the ileal metabolomic profile but selectively alleviates heat stress-associated metabolic disturbances, particularly those related to amino acid homeostasis, redox regulation, and carbon metabolism.

### Associations between TCM-alleviated metabolites and physiological indicators under acute and chronic heat stress

**Fig. 9:**
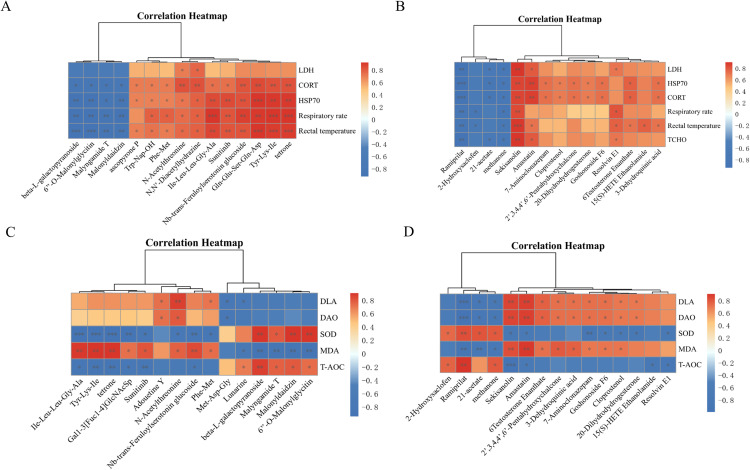
Correlation analysis between traditional Chinese medicine (TCM)-alleviated metabolites and physiological, stress, and intestinal parameters under heat stress. (A-B) Correlation between TCM-alleviated metabolites and physiological and stress indicators under AHS (A) and CHS (B). (C-D) Correlation between TCM-alleviated metabolites and intestinal barrier and oxidative status markers under AHS (C) and CHS (D). RT: rectal temperature, RR: respiratory rate, LDH: lactate dehydrogenase, HSP70: heat shock protein 70, CORT: corticosterone, TCHO: total cholesterol, SOD: superoxide dismutase, MDA: malondialdehyde, T-AOC: total antioxidant capacity, DLA: d-lactic acid, DAO: diamine oxidase.

Correlation analyses were conducted to explore the associations between TCM-alleviated metabolites and key physiological indicators related to thermoregulation and stress responses. Although the sets of metabolites differed between the TCM.AHS and TCM.CHS groups, metabolites from both conditions showed significant correlations with heat stress-associated parameters, including rectal temperature, respiratory rate, lactate dehydrogenase (LDH), heat shock protein 70 (HSP70), and corticosterone (CORT) (Fig. 9A-B). A greater proportion of metabolites alleviated under TCM.AHS were significantly correlated with rectal temperature and respiratory rate, indicating a closer association between acute-phase metabolic modulation and physiological heat-stress responses (Fig. 9A). In contrast, metabolites alleviated under TCM.CHS displayed more diverse correlation patterns with systemic metabolic indicators. Notably, specific metabolites, including ramiprilat and sekisanolin, were significantly correlated with total cholesterol (TCHO), suggesting that chronic heat stress-associated metabolic modulation may influence lipid metabolism (Fig. 9B).

Further correlation analyses with intestinal barrier and oxidative status markers showed that metabolites alleviated under TCM.AHS were predominantly associated with antioxidant parameters in the ileum, including superoxide dismutase (SOD), malondialdehyde (MDA), and total antioxidant capacity (T-AOC) (Fig. 9C). Conversely, metabolites alleviated under TCM.CHS showed significant correlations with both intestinal barrier indicators, such as d-lactate and diamine oxidase (DAO), and antioxidant parameters (Fig. 9D). Collectively, these findings suggest that TCM-mediated metabolic modulation under acute heat stress is closely linked to immediate thermoregulatory and oxidative responses, whereas under chronic heat stress, it is associated with coordinated alterations in antioxidant capacity and intestinal barrier function.

## Discussion

In the present study, heat stress triggers profound physiological, metabolic, and productive disturbances in laying hens, with these responses evolving significantly from the acute to the chronic stage. Both acute (6 h) and chronic (6 h, 14 d) exposure to 36°C resulted in immediate systemic stress, characterized by significant elevations in rectal temperature, respiratory rate, and serum stress indicators such as corticosterone and HSP70. These findings align with previous reports indicating that ambient temperatures exceeding the thermoneutral zone severely compromise the thermoregulatory capacity and endocrine balance of poultry (Kim, et al., 2024; Oluwagbenga and Fraley, 2023). Furthermore, the observed reductions in egg production and eggshell strength are consistent with previous reports identifying heat-induced systemic alterations and limited nutrient availability as primary drivers of reproductive impairment in laying hens (Nanto-Hara, et al., 2023; Wasti, et al., 2020).

A critical observation in this research is the distinct stage-dependent response between acute and chronic heat stress. While acute exposure induces rapid oxidative damage and transient metabolic perturbations, such as elevated glucose and reduced total cholesterol, chronic exposure leads to broader metabolic remodeling and physiological adjustment. Specifically, the partial recovery of serum total cholesterol during chronic exposure suggests a systemic adjustment to prolonged thermal challenge, representing a transition from immediate stress responses to a state of sustained metabolic conservation. This is consistent with the principle of physiological recalibration, where long-term environmental stress induces a shift in systemic metabolism to ensure survival at the expense of productive efficiency (Baumgard and Rhoads, 2013; Kim, et al., 2024). Our metabolomic findings further support this temporal divergence, demonstrating that acute stress primarily impacts amino acid pathways associated with immediate response, whereas chronic stress leads to more extensive alterations in energy utilization and one-carbon metabolism. Unlike many studies that focus on a single duration of heat exposure, our comparative analysis highlights the importance of considering the duration of thermal challenge when evaluating host adaptive strategies and intervention efficacy (An, et al., 2025; Kikusato and Toyomizu, 2023; Xie, et al., 2015).

Dietary supplementation with a compound traditional Chinese medicine (TCM) formulation represents an effective nutritional strategy to enhance heat resilience in laying hens. Our findings demonstrate that 0.5% TCM supplementation exerts multi-target protective effects, ranging from the restoration of systemic physiological homeostasis to the preservation of local intestinal integrity. These results align with previous studies indicating that herbal additives can improve poultry health by modulating immune and antioxidant systems (Fayed, et al., 2024; Li, et al., 2024; Oni, et al., 2024; Ravi, et al., 2025). Notably, the efficacy of TCM exhibited distinct stage-dependent characteristics: while it provided immediate buffering against acute oxidative damage, its restorative effects on hormonal balance and productive performance were more pronounced during the chronic phase. By integrating multiple datasets, this study clarifies that the protective role of TCM is primarily driven by the reprogramming of ileal metabolic pathways, which are supported by corresponding shifts in the gut microbial community.

The mitigation of systemic stress indicators is a primary mechanism by which TCM preserves productive performance, with varying efficacy across stress durations. High ambient temperatures activate the hypothalamic-pituitary-adrenal (HPA) axis, leading to elevated serum corticosterone (CORT), which inhibits yolk precursor synthesis and compromises follicular development (Kim, et al., 2024). We observed that while TCM attenuated CORT and HSP70 elevations during acute heat stress (AHS), it completely restored these indicators to pre-stress levels under chronic heat stress (CHS). This suggests that TCM facilitates a more thorough neuroendocrine recalibration during prolonged exposure compared to its immediate response during acute shock. This stabilization likely underpinned the significant recovery in daily laying rate and eggshell quality observed during the latter stage of CHS. This is consistent with the findings, who reported that herbal components could stabilize the endocrine environment, though our results further highlight that this recovery is particularly effective during sustained thermal exposure (Greene, et al., 2024; Onagbesan, et al., 2023; Wasti, et al., 2020).

The intestine is the primary bottleneck for poultry health under heat stress, serving as a site for both immediate oxidative injury and chronic inflammatory remodeling (Ncho, 2025; Rostagno, 2020). Our results indicate that TCM protected the ileal barrier through stage-specific molecular mechanisms. Under AHS, TCM primarily improved crypt depth and upregulated tight junction proteins *ZO-1* and *CLDN1*, likely to counteract the rapid increase in epithelial permeability. In contrast, during CHS, the protective effect shifted towards enhancing enzymatic defenses, such as significantly increasing SOD activity, which was not observed during the acute phase. This structural and biochemical preservation is consistent with the protective effects of herbal polysaccharides reported previously (Liu, et al., 2023).

At the microbial level, the consistent recovery of specific taxa, such as members of the genus *Olsenella*, serves as a representative indicator of the functional restoration of the gut ecosystem. While many studies suggest that heat stress primarily depletes Lactobacillus, our findings suggest that TCM maintains a core microbial community that aligns with the host’s metabolic needs (Lyte, et al., 2025; Ncho, 2025). Taxa such as *Olsenella uli* likely represent a broader microbial shift toward supporting carbohydrate fermentation and energy production. The enrichment of starch and sucrose metabolism in these microbial groups suggests that they provide a stable functional foundation that complements the host’s metabolic adjustments. The strong correlation between these microbial features and reduced rectal temperature supports the hypothesis that a stable microbial environment is a necessary collaborator in the host’s overall thermoregulatory resilience.

The metabolomic reprogramming observed in this study serves as the central mechanism for TCM-mediated heat resilience. Under AHS, TCM primarily modulated tryptophan metabolism, a pathway often exhausted during sudden stress due to increased antioxidant and immune demands (Li, et al., 2023). Tryptophan-derived metabolites are essential for acute immune regulation and mitigating immediate oxidative damage. Conversely, during CHS, TCM uniquely influenced the one-carbon pool by folate, pantothenate/CoA biosynthesis, and betaine metabolism. The one-carbon cycle is indispensable for nucleotide synthesis and methylation reactions required for DNA repair and cellular regeneration after prolonged thermal injury (Ducker and Rabinowitz, 2017; Locasale, 2013). This metabolic shift indicates that the primary protective role of TCM lies in its ability to facilitate a transition from acute stress-buffering to long-term metabolic stability and tissue repair, providing the necessary biochemical precursors for sustained physiological recalibration.

Despite these promising findings, some limitations warrant consideration. While we identified specific microbial and metabolic biomarkers, the direct causal interactions within the microbiota-metabolome axis require further validation through targeted intervention studies. Additionally, the complex synergy between the twelve herbal ingredients in the TCM formulation requires further investigation to identify the primary bioactive compounds responsible for stage-specific metabolic reprogramming. In conclusion, this study demonstrates that TCM supplementation is a viable precision nutritional approach to improve heat tolerance in laying hens by primarily modulating ileal metabolic functions and stabilizing the gut microbial environment, offering a sustainable strategy to mitigate both acute and chronic thermal challenges in the poultry industry.

## Conclusion

Dietary supplementation with a 0.5% compound traditional Chinese medicine (TCM) formulation serves as a highly effective nutritional intervention to sustain the productive performance of laying hens under both acute and chronic heat stress. Our findings demonstrate that TCM significantly mitigates heat-induced declines in daily laying rate, egg weight, and eggshell strength, particularly during prolonged thermal exposure. These improvements in productivity are fundamentally supported by the restoration of systemic physiological homeostasis and the preservation of intestinal barrier integrity. The central mechanism involves a stage-specific reprogramming of the ileal metabolome—specifically modulating tryptophan metabolism to provide immediate antioxidant protection during acute stress, while supporting the one-carbon pool and energy-related biosynthesis to facilitate tissue repair and metabolic stability during chronic exposure. Collectively, these results provide evidence that TCM is a viable precision nutritional strategy to optimize productive efficiency and protect animal welfare in commercial poultry operations facing increasing environmental challenges.

## CRediT authorship contribution statement

**Zi Mei:** Writing – review & editing, Writing – original draft, Software, Investigation, Data curation. **Haobo Zhou:** Writing – review & editing, Software, Methodology. **Kunyuan Liu:** Project administration, Investigation. **Chaoyang Gao:** Project administration. **Hao Du:** Project administration. **Zheya Sheng:** Writing – review & editing, Writing – original draft, Resources, Funding acquisition. **Yanzhang Gong:** Writing – review & editing, Writing – original draft, Resources, Funding acquisition.

## Disclosures

The authors declare no conflicts of interest.

## Appendix Supplementary materials

Image, application 1

## Footnotes

Supplementary material associated with this article can be found, in the online version, at doi:10.1016/j.psj.2026.107056.
