# Supplementary material for: Traditional Chinese medicine improves performance and intestinal health in laying hens under acute and chronic heat stress by modulating ileal metabolic functions
Source: Poult Sci. 2026 May 1;105(8):107056. doi: 10.1016/j.psj.2026.107056 (PMC13196068; doi:10.1016/j.psj.2026.107056)
Supplement: Supplementary file 1 [file mmc1.docx]

|  | CON | TCM | AHS | TCM.AHS | CON | TCM | CHS | TCM.CHS |
| --- | --- | --- | --- | --- | --- | --- | --- | --- |
| Duodenum |  |  |  |  |  |  |  |  |
| Villus height (μm) | 1612.47±177.18^b^ | 1683.00±156.53^b^ | 1302.03±227.66^a^ | 1774.05±145.36^b^ | 1579.21±107.26 | 1554.11±176.90 | 1685.36±157.86 | 1701.95±43.01 |
| Crypt depth (μm) | 218.89±48.17 | 206.32±38.64 | 217.89±19.25 | 227.75±61.62 | 207.61±17.44 | 201.61±37.34 | 178.14±20.27 | 202.76±20.56 |
| Villus height/crypt depth  (μm/μm) | 7.95±1.90 | 8.55±1.99 | 6.17±1.34 | 8.34±1.98 | 7.87±0.83 | 8.15±2.48 | 9.63±1.46 | 9.06±1.20 |
| Jejunal |  |  |  |  |  |  |  |  |
| Villus height (μm) | 1020.62±279.68 | 1188.03±267.93 | 1045.38±215.85 | 1099.93±185.99 | 1116.26±201.10 | 1389.57±224.52 | 1127.37±152.37 | 1298.25±272.75 |
| Crypt depth (μm) | 164.32±50.82 | 158.73±33.51 | 189.79±52.65 | 171.77±16.71 | 129.37±21.89 | 173.65±56.40 | 160.7±37.23 | 207.47±56.80 |
| Villus height/crypt depth  (μm/μm) | 6.45±1.08^b^ | 7.77±0.99^a^ | 5.73±0.99^b^ | 6.47±0.49^b^ | 8.94±1.85 | 8.62±2.70 | 7.54±2.50 | 6.90±2.95 |

Table S1 Pathological Analysis of Jejunum and Ileum Sections

Notes: Values are presented as mean ± SEM. Different lowercase letters (a–c) within the same row indicate significant differences among treatment groups at the same time point (*P < 0.05*).


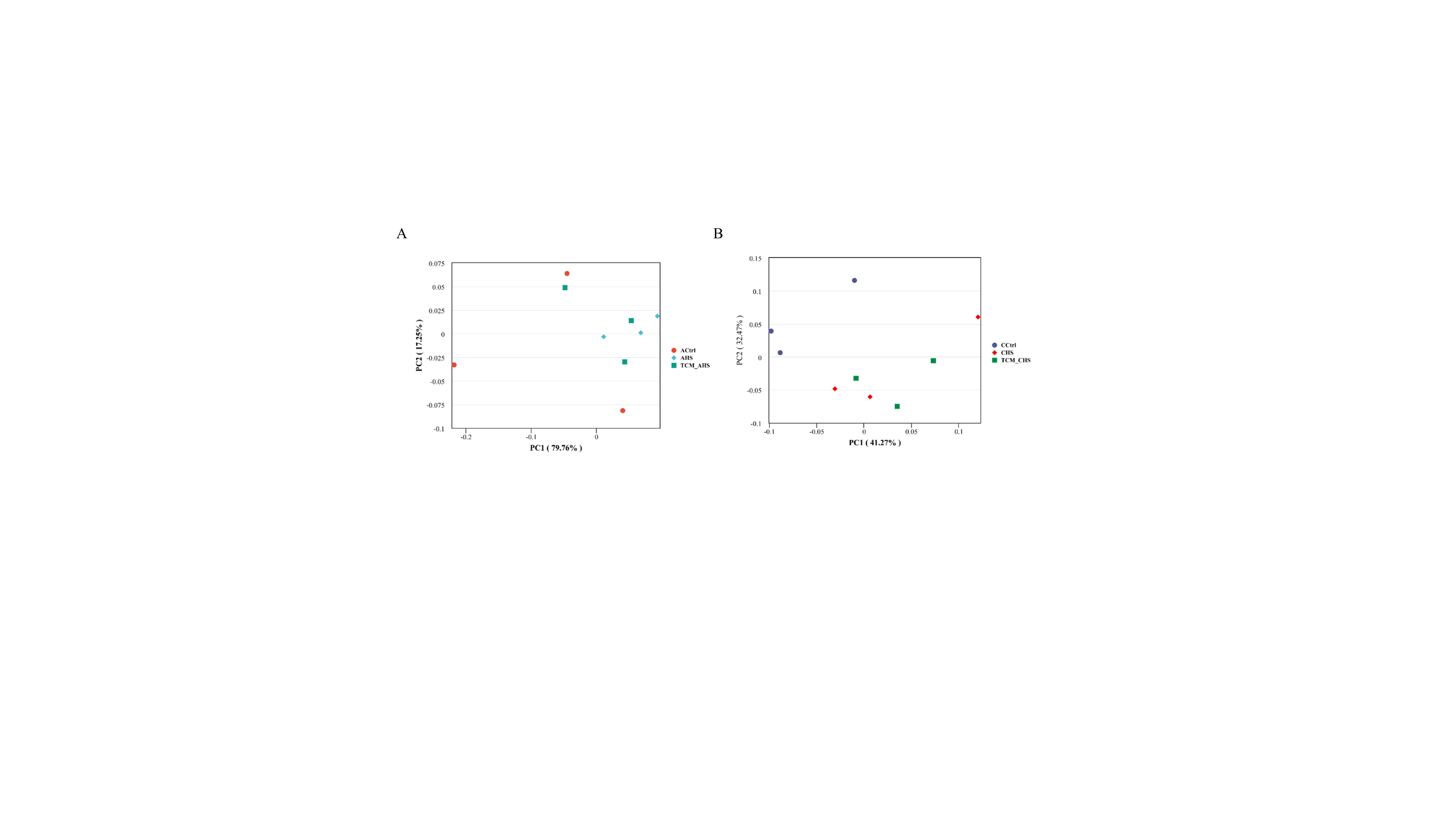


Fig. S1. Principal component analysis (PCA) of the ileal microbial community under acute and chronic heat stress. PCA based on species abundance showing the distribution of ileal microbiota in different treatment groups. (A) PCA score plot under acute heat stress (AHS, 6 h). (B) PCA score plot under chronic heat stress (CHS, 14 d).


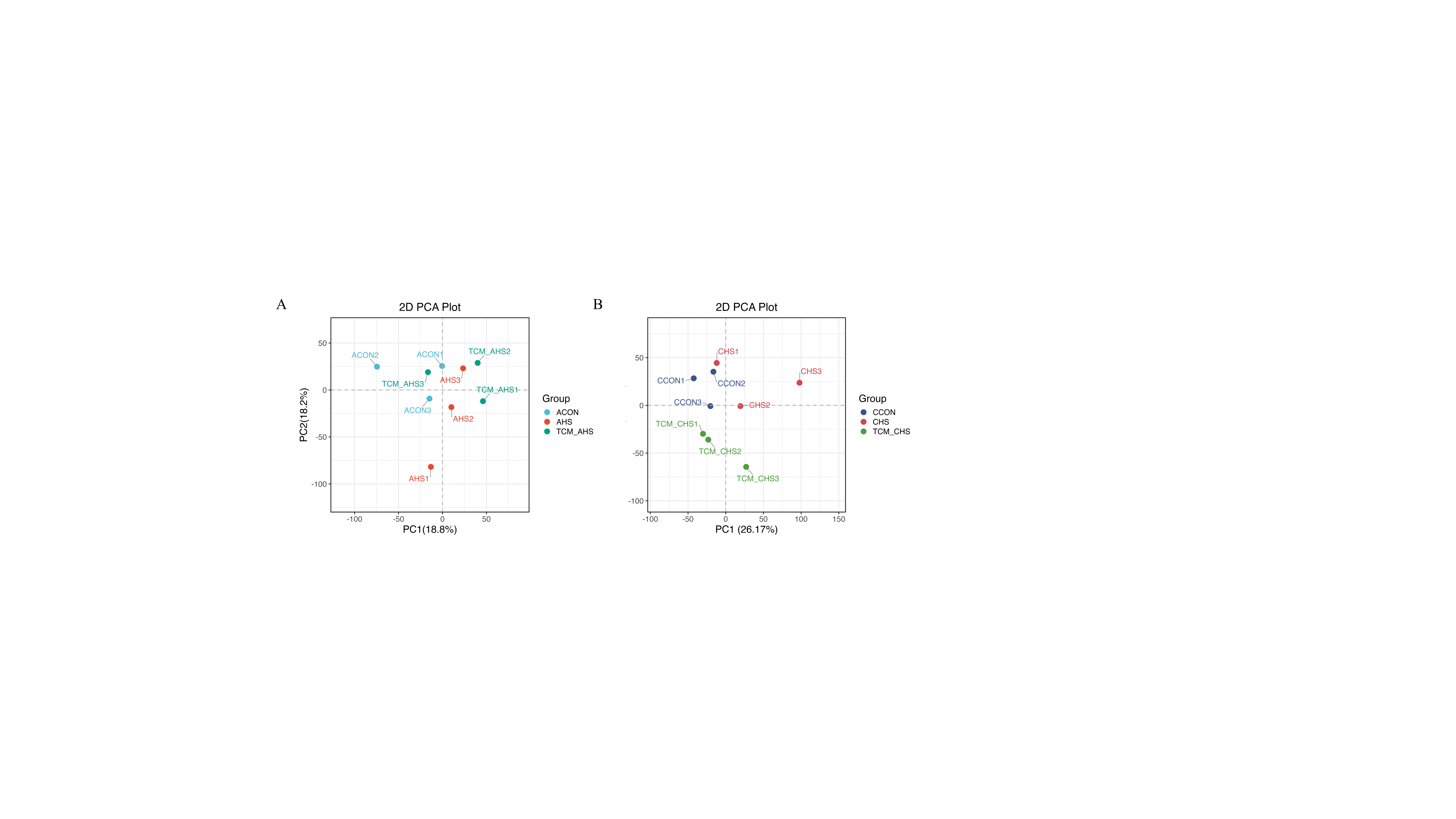


Fig. S2. Principal component analysis (PCA) of ileal metabolite profiles under acute and chronic heat stress conditions. PCA score plots illustrate the overall distribution and separation of ileal metabolomic profiles among different treatment groups. (A) PCA score plot under acute heat stress (AHS, 6 h of heat exposure). (B) PCA score plot under chronic heat stress (CHS, 14 d of heat exposure).
